# Treatment of Hemorrhoids with Nitric Acid

**Published:** 1850-01

**Authors:** S. Weed

**Affiliations:** Port Byron, N. Y.


### ARTICLE IV.

*Hemorrhoids Treated by Nitric Acid.*—By S. Weed, M.D., of Port Byron, N. Y. (From the Buffalo Med. Journal.)

Dr. Flint:—Having had under my care a patient afflicted for a good many years with hemorrhoids of an aggravated character, the treatment of which, under my hands, has been most eminently successful—much more so than I had anticipated when I commenced it, I have deemed it incumbent on me to report the same for your Journal, that the profession may be able to judge as to its applicability to similar cases.—The following is from notes taken during the progress of the treatment:—

May 15th—Was called into the country to-day, to see a Mrs. J., evidently in the last stages of anæmia, induced by hemorrhoids. Mrs. J. is a married woman, and has three or four children. Has been troubled with piles ever since, and before she was married. Has leucorrhœa, also, which difficulty she has been laboring under for a long time. Is or rather feeble constitution. Hemorrhage amounts to from two to eight ounces per day, I should think, from accounts given by both Mr. and Mrs. J. Lips and tongue are exsanguineous; dark circle about her eyes. Countenance has a cadaverous hue; patient confined to her bed, and on the least exertion, her heart is thrown into violent fits of palpitation; hands and feet are cold, accompanied with frequent chills. Mrs. J. has been treated by a great number and variety of physicians. Former treatment has been of but little avail. Did not examine the parts to-day. Told the friends that I was not prepared to do much to-day. Ordered tonics and stimulants until I should see her again next day.

May 16th.—Visited Mrs. J. to-day, and found that my prescription had been successful in keeping her warm. Made an examination. Tumors about as large as a medium sized tomato. Blood was oozing from the exposed tumors. Great deal of difficulty experienced in voiding the *fæces*.

*Treatment.*—Had resolved upon cauterization. Made choice of nitric acid. Made a free application of the acid to the exposed tumors. Pain not as severe as I had anticipated, from the application. Patient said that she had frequently experienced more pain while at stool, than from the caustic.—Dressed the parts with a cloth moistened with olive oil. Ordered muriate of iron in sweet spirits of nitre, to correct anemia. Did not visit patient on the 17th.

May 18th.—Found Mrs. J. tolerably comfortable. Had experienced considerable pain the night following the application of the acid. Found the parts somewhat swollen. There had been no hemorrhage, although the patient had had three motions of the bowels since my last visit. Discharge considerable from the cauterized surface. This was quite purulent. She stated that she had experienced scarcely any trouble in voiding the fæces. Ordered simple bitters, in connection with the muriate. Also applied cloths wet with laudanum, to the tumors. After she should become quiet, or free from pain, told her to make cold applications.

May 19th.—No hemorrhage, as yet. Patient quite feeble. Less difficulty and uneasiness in voiding the *fæces*. Less discharges of pus than on the day before. Some difficulty in passing urine, accompanied by considerable pain in the loins.

Ordered sweet spirits of nitre every three or four hours, until relief should be experienced. The tinct. opii, ordered the day before, had been of much service by way of relieving pain.

May 20th.—Found Mrs. J. improving. She felt, and looked much better than at any time since I had commenced visiting her. Had a very good appetite, for the first, for a long time. Was quite cheerful. No hemorrhage. Scarcely any difficulty in voiding the fæces. No difficulty in passing urine. Ordered ungt. hyd. mitius to be applied twice a day. Tonics as before.

May 21st.—Patient still improving. No hemorrhage. Very little irritation remaining. Tumors diminishing in size. Appetite quite good. Bowels somewhat constipated. Pulse more full, and less frequent. Ordered sulphur and bitartrate of potassa in molasses, to relieve constipation. Stopped the use of the ungt.

It is unnecessary for me to relate the progress made by my patient from day to day, until I dismissed her. I attended Mrs. J. some three weeks. She had no return of the hemorrhage. Her appetite remained good. When I dismissed her, she told me that all the tumor there was remaining, was one about the size of a chestnut. She had then so far recovered her health, that she was able to be about the house, and see to her own work. During the latter part of the treatment, I ordered injections of a solution of sulph. ferri, for the leucorrhea. For the last week of my attendance, I changed the muriate of iron to the carbonate, and quinine. I have seen Mrs. J. a number of times since I attended upon her, and have found her able to attend to her domestic concerns, to do her own work, to go to the neighbors, &c., &c.

The above case I consider the more interesting from the fact that it had resisted every other treatment but the one I adopted.

I am aware that the treatment which I adopted in the above case, is not altogether new. Various writers speak of it.—But is it not too much neglected? Would not the treatment by caustics, if generally followed in severe cases, be much more satisfactory than that by astringents, &c?

*October* 26*th*, 1849.

